# Seed germination strategies: an evolutionary trajectory independent of vegetative functional traits

**DOI:** 10.3389/fpls.2015.00731

**Published:** 2015-10-12

**Authors:** Gemma L. Hoyle, Kathryn J. Steadman, Roger B. Good, Emma J. McIntosh, Lucy M. E. Galea, Adrienne B. Nicotra

**Affiliations:** ^1^Department of Evolution, Ecology and Genetics, Research School of Biology, Australian National UniversityCanberra, ACT, Australia; ^2^School of Pharmacy and Queensland Alliance for Agriculture and Food Innovation, The University of QueenslandQLD, Australia; ^3^Australian National Botanic GardensCanberra, ACT, Australia; ^4^Fenner School of the Environment, Australian National UniversityCanberra, ACT, Australia

**Keywords:** alpine plants, climate change, dormancy, endosperm, germination strategy, phylogenetic regression

## Abstract

1. Seed germination strategies vary dramatically among species but relatively little is known about how germination traits correlate with other elements of plant strategy systems. Understanding drivers of germination strategy is critical to our understanding of the evolutionary biology of plant reproduction.
2. We present a novel assessment of seed germination strategies focussing on Australian alpine species as a case study. We describe the distribution of germination strategies and ask whether these are correlated with, or form an independent axis to, other plant functional traits. Our approach to describing germination strategy mimicked realistic temperatures that seeds experience *in situ* following dispersal. Strategies were subsequently assigned using an objective clustering approach. We hypothesized that two main strategies would emerge, involving dormant or non-dormant seeds, and that while these strategies would be correlated with seed traits (e.g., mass or endospermy) they would be largely independent of vegetative traits when analysed in a phylogenetically structured manner.
3. Across all species, three germination strategies emerged. The majority of species postponed germination until after a period of cold, winter-like temperatures indicating physiological and/or morphological dormancy mechanisms. Other species exhibited immediate germination at temperatures representative of those at dispersal. Interestingly, seeds of an additional 13 species “staggered” germination over time. Germination strategies were generally conserved within families. Across a broad range of ecological traits only seed mass and endospermy showed any correlation with germination strategy when phylogenetic relatedness was accounted for; vegetative traits showed no significant correlations with germination strategy. The results indicate that germination traits correlate with other aspects of seed ecology but form an independent axis relative to vegetative traits.

## Introduction

The timing of seed germination dictates a seedling's seasonal exposure to potentially lethal environmental factors, and thus has strong fitness consequences (Simons and Johnston, 2000; Donohue, 2005). However, for much of the world's flora the particular mechanisms that regulate seasonal emergence patterns are unknown. These mechanisms may include a combination of environmental germination requirements and seed dormancy. Given the importance of germination timing these traits are likely to evolve in correlated suites with other key functional traits. However, it is unclear whether germination strategy is correlated with other axes of plant strategy (e.g., seed mass or leaf mass per unit area), or indeed constitutes an additional independent axis. In the context of a rapidly changing climate, understanding the germination strategies of native species from threatened communities moves from being a question of evolutionary and ecological interest, to an urgent matter for conservation and management goals.

Seed mass declines with increasing latitude (Moles and Westoby, 2004) and has been shown to be correlated with a range of traits, including early seedling survival in low light, growth form, and dispersal syndrome (Leishman et al., 1995; Westoby et al., 1996; Moles et al., 2007). However, little is known about whether other reproductive traits, including germination strategies, correlate with other seed traits, with leaf or whole plant traits, or whether they might form another independent axis entirely.

Seed dormancy mechanisms are regarded as the principle means by which seeds can control the timing of germination and thus are expected to be under strong selective pressure. Dormant seeds sense and respond to their environment (Vleeshouwers et al., 1995) in order to avoid a germination response to temperature or rainfall that would not support subsequent seedling growth (Tielborger et al., 2012). Dormancy may result from physical, physiological or developmental/morphological mechanisms, or combinations thereof (Baskin and Baskin, 2001). In understanding dormancy, however, documenting the presence of a dormancy mechanism is just the first step: understanding the role of that dormancy mechanism in controlling the timing of germination is a crucial step. Studies of germination strategy frequently bypass or terminate dormancy through the application of chemical agents for logistical reasons (Cohn et al., 1989; Foley, 1992), but doing so reveals little about when it is alleviated naturally, *in situ*. In contrast, investigating germination strategies under ecologically relevant experimental conditions that mimic seasonal temperature regimes and seed moisture content can alleviate dormancy in a way that reveals much more about innate germination strategies (Baskin and Baskin, 2004; Albrecht and McCarthy, 2006; Hoyle et al., 2008a).

Climatically extreme environments, such as alpine and high montane regions, are characterized by spatially variable and temporally unpredictable conditions, particularly low temperatures and short growing seasons (Bliss, 1971; Körner, 2003). Conditions for seedling establishment may not be favorable for all species immediately after seeds are dispersed or even during the subsequent growing season, nor will species be equally equipped to cope with winter conditions during early seedling establishment. Therefore, seeds of species found in alpine environents are expected to vary in germination traits and strategies (Wagner and Simons, 2009). Seed dormancy was once considered relatively rare among alpine plant species (Amen, 1966), however, more recent studies indicate physiological dormancy may control the timing of germination in alpine systems more often than previously thought (Densmore and Zasada, 1983; Cavieres and Arroyo, 2000; Shimono and Kudo, 2005; Mondoni et al., 2009; Sommerville et al., 2013). Thus, the alpine flora provides an ideal context in which to assess the evolution of germination strategy.

When the ecology of plant species is described from the perspective of functional traits it becomes apparent that some traits form correlated suites that are robust whether considered at an absolute scale or in a phylogenetically controlled design. For example, the leaf economic spectrum (LES) describes a continuum of strategies ranging from slow growth and high cost leaves to rapid growth potential, short lived leaves and high photosynthetic rates (Reich et al., 1997; Wright et al., 2004). The LES can be viewed in an extended form as a whole plant strategy for water or carbon use (Reich, 2014). But in some cases traits sort more effectively into suites that form independent axes. For example, seed mass, mature plant height and leaf mass per unit area show little intercorrelation (Westoby, 1998). It remains to be determined how other aspects of seed ecology, particularly germination strategy, correlate with functional traits such as seed mass and vegetative characteristics.

The present study investigated germination strategies and ecological correlates thereof in 54 Australian species from 16 families, including 10 endemics and include a range of species from grassland specialists, to widely distributed species also found in bogs, fens and shrublands. We hypothesized that species would exhibit one of two possible germination strategies: pre-winter germination of non-dormant seeds or postponed germination via dormancy mechanisms and that these would show conservation among families. Further, we asked whether germination strategy would be correlated with other ecological traits such that species that germinated immediately would also show traits of opportunistic growth (e.g., higher leaf mass per unit area, shorter mature heights and/or smaller seed mass), or whether germination strategy would comprise an independent axis to these functional traits.

## Materials and methods

### Study site

**Figure 1:**
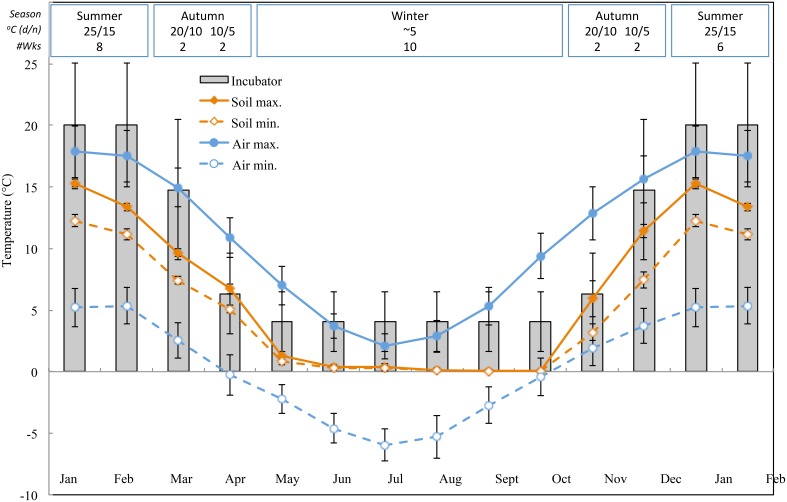
**Monthly maximum and minimum air and soil temperature data in the Australian Alps (mean ± STDEV)**. Air temperatures are averages from the BOM database, soil temperatures were measured with iButtons (see methods). Bars indicate experimental incubator temperatures (mean ± STDEV). The equivalent time of year (season), incubator temperature regime (°C, 12/12 h, light/dark) and duration of treatment (weeks) as indicated in the top panel.

The Australian Alps are located in southeast Australia and cover approximately 25000 km^2^. Seed collections were made at altitudes ranging from 1605 to 2212 m a.s.l. in the New South Wales portion of Kosciuszko National Park and including high elevation frost hollows as well as true alpine sites above treeline. Seeds were collected from herbfields and grasslands incorporating a range of bog and fen habitats and a mix of specialist and generalist species distributed along moisture gradients. More than half of Kosciuszko National Park's annual rainfall (1800–3100 mm) falls as winter snow and persists for at least 4 months. Data collected by the Bureau of Meteorology at Charlotte Pass (Kosciuszko Chalet; 36.43°S, 148.33°E, 1755 m a.s.l.) in Kosciuszko National Park between 1968 and 2014 indicate that air temperatures are commonly below zero during winter months and average between 15 and 20°C during summer (Bureau of Meteorology, 2010, http://www.bom.gov.au. Figure 1).

With a view to uncovering temperature conditions close to those that seeds experience post-dispersal, daily maximum and minimum soil temperature data were collected within the study site (Seaman's Hut; 36.27°S and 148.17°E, 2030 m a.s.l.) using ibutton data loggers (*n* = 10, Embedded Data Systems, USA) placed 4 cm below the soil surface at the base of vegetation from 17 January to 17 December 2012. We placed loggers at 4 cm to avoid surface disturbance but still be representative of conditions to which seeds are exposed in soil; this depth is intermediate to what has been used in prior alpine soil temperature monitoring exercises (Scherrer and Körner, 2011; Pauli et al., 2015). As suspected, average soil temperature did not drop below freezing during winter (Figure 1). In summer, soil temperature under vegetation is known to track ambient temperature, whereas temperatures of bare soil will exceed ambient by up to 15°C (Soil Conservation Service unpublished records, 1960s–1970s).

### Seed collecting and germination

Mature seeds of 54 species from 16 families and 37 genera were collected between January and April 2009, 2010, and 2011 (see Table 1 for full names and authorities. Vouchers were lodged at the Australian National Herbarium, Canberra). In total the species represented more than a quarter of the Australian angiosperm flora found in alpine regions (Costin et al., 2000, see Table 1), though many of these species extend to below treeline as well. The viability of all collections was estimated prior to sowing in experimental germination conditions using the tetrazolium chloride (TZ) staining technique (International Seed Testing Association, 2003). For more details on collection and processing see Appendix A in Supplementary Material.

**Table 1:** **Study species, family and authority, growth form, and collections details, percentage seed viability (TZ-estimated, ± s.e.), final percentage germination achieved (± s.e.), statistical difference between viability and germination (G < V, tested only in cases where germination was less than viability)**.

**Family**	**Species**	**Growth form**	**Date collected**	**Elev (m a.s.l.)**.	**Viability (%, SE)**		**Germination (%, SE)**		**G < V**	**Cluster**	**Accession no.**
Apiaceae	*Aciphylla glacialis* (F.Muell.) Benth.	Herb	11-Mar-09	2164	100	0	95	3.8	ns	Postponed	CANB 782521
	*Aciphylla glacialis* (F.Muell.) Benth.	Herb	17-Feb-10	1937	85	4.8	91	2.1		Postponed	CANB 792173
	*Aciphylla simplicifolia* (F.Muell.) Benth.	Herb	12-Mar-09	1698	100	0	81	5.9	^*^	Postponed	CANB 782524
	*Aciphylla simplicifolia* (F.Muell.) Benth.	Herb	16-Feb-10	1743	85	15	80	3	ns	Postponed	CANB 792163
	*Diplaspis nivis* Van den Borre and Henwood	Herb	28-Jan-10	1960	64	16	88	3.1		Postponed	CANB 786949
	*Gingidia algens* (F.Muell.) J. W. Dawson^†^	Herb	16-Feb-10	1740	97	3.3	94	1.2	ns	Postponed	CANB 792164
	*Oreomyrrhis ciliata* Hook.f.	Herb	24-Mar-09	1748	92	8.3	94	2.6		Staggered (A2)	CANB 783452
	*Oreomyrrhis ciliata* Hook.f.	Herb	27-Jan-10	1748	100	0	100	0		Staggered (A3)	CANB 792149
	*Oreomyrrhis eriopoda* (DC.) Hook.f.	Herb	24-Feb-09	1743	97	2.8	93	3.1	ns	Staggered (A2)	CANB 783437
	*Oreomyrrhis eriopoda* (DC.) Hook.f.	Herb	3-Feb-10	1744	83	12	54	9	ns	Postponed	CANB 792151
	*Oreomyrrhis pulvinifica* F.Muell.	Herb	4-Feb-10	1733	77	8.8	88	2.9		Staggered (A3)	CANB 786952
	*Schizeilema fragoseum* (F.Muell.) Domin	Herb	31-Mar-11	2092	67	6.7	1	1	^***^	too low to score	CANB 798462
Asteraceae	*Brachyscome obovata* G. L. R. Davis	Herb	2-Mar-11	2090	77	5.5	65	3.1	ns	Immediate	CANB 797826
	*Brachyscome stolonifera* G. L. Davis^†^	Herb	5-Feb-10	1941	70	15	22	6.4	^*^	Staggered (B2)	CANB 792161
	*Celmisia costiniana* M. Gray and Given	Herb	2-Mar-11	2046	61	3.2	90	3.5		Staggered (B2)	CANB 798295
	*Craspedia jamesii* J. Everett and Joy Thomps.	Herb	1-Feb-11	1730	70	10	78	3.6		Immediate	CANB 797734
	*Craspedia lamicola* J. Everett and Joy Thomps.	Herb	3-Mar-11	2067	93	3.3	93	2.1	ns	Immediate	CANB 798305
	*Craspedia leucantha* F.Muell.^†^	Herb	25-Feb-09	1996	93	3.7	96	1.6		Immediate	CANB 783441
	*Erigeron bellidioides* (Hook.f.) S. J. Forbes and D. I. Morris	Herb	29-Jan-10	1730	83	6.7	95	1.9		Immediate	CANB 792150
	*Erigeron nitidus* S. J. Forbes	Herb	17-Feb-10	2007	70	5.8	93	4.3		Immediate	CANB 792172
	*Erigeron setosus* (Benth.) M.Gray^†^	Herb	4-Feb-10	1928	57	1.5	100	0		Immediate	CANB 792158
	*Leucochrysum alpinum* (F.Muell.) R. J. Dennis and N. G. Walsh	Mat/ Herb	11-Mar-09	2212	77	3.3	100	0		Immediate	CANB 782523
	*Ozothamnus cupressoides* Puttock and D. J. Ohlsen	Shrub	9-Apr-09	1605	82	19	21	13.8	^*^	Staggered (A3)	CANB 782558
Campanulaceae	*Wahlenbergia ceracea* Lothian	Herb	3-Feb-10	1744	87	7.2	78	10.6	ns	Staggered (A2)	CANB 792185
Caryophyllaceae	*Colobanthus affinis* (Hook.) Hook.f.	Herb	25-Mar-09	2069	90	5.8	95	3.9		Immediate	CANB 782529
	*Colobanthus affinis* (Hook.) Hook.f.	Herb	5-Jan-10	1736	93	3.3	100	0		Immediate	CANB 786943
	*Scleranthus biflorus* (J. R. Forst. and G. Forst.) Hook.f.	Mat/ Cushion	10-Mar-09	1751	77	6.5	82	3.6		Immediate	CANB 782512
Cyperaceae	*Carex cephalotes* F.Muell.	Sedge	4-Jan-09	1613	77	5.5	78	5.5		Postponed	CANB 771067
	*Carex cephalotes* F.Muell.	Sedge	5-Jan-10	1640	96	3.7	93	2.3	ns	Postponed	CANB 771067
	*Carex echinata* Murray	Sedge	21-Jan-09	1684	83	11	98	1.5		Postponed	CANB 783404
	*Carex echinata* Murray	Sedge	3-Feb-10	1742	83	3.3	98	2.1		Postponed	CANB 792155
	*Carpha nivicola* F.Muell.	Sedge	3-Feb-10	1730	86	8.8	84	5.9	ns	Postponed	CANB 792153
	*Oreobolus pumilio* R.Br.	Sedge	7-Apr-09	1945	80	5.5	81	8.9		Postponed	CANB 782540
	*Uncinia flaccida* S. T. Blake^∧^	Sedge	3-Mar-11	2047	97	3.3	13	15.7	^***^	Postponed	CANB 798306
Droseraceae	*Drosera arcturi* Hook.	Herb	27-Jan-10	1752	97	3.3	98	1.2		Postponed	CANB 786948
Ericaceae	*Epacris petrophila* Hook.f.	(Sub) shrub	26-Mar-09	1745	71	5.8	93	3.2		Postponed	CANB 782533
	*Pentachondra pumila* (J. R. Forst. and G. Forst.) R.Br.^∧^	Mat/Sub -shrub	17-Feb-10	2096	85	8.3	27	4.3	^***^	Postponed	CANB 792168
	*Richea continentis* B. L. Burtt	(Sub) shrub	8-Apr-09	2078	83	3.8	95	1.1		Postponed	CANB 782545
Gentiananaceae	*Gentianella muelleriana subsp. alpestris* (L. G. Adams) Glenny^†^	Herb	24-Mar-09	1748	62	9.6	74	3.8		Postponed	CANB 783451
	*Gentianella muelleriana subsp. alpestris* (L. G. Adams) Glenny^†^	Herb	30-Mar-11	2109	83	3.8	1	1	^***^	too low to score	CANB 798460
Juncaceae	*Juncus falcatus* E.Mey.	Rush	10-Mar-09	1742	64	4.7	54	15.8	ns	Postponed	CANB 782513
	*Luzula acutifolia* subsp. *nana* Edgar^†^	Rush	25-Feb-09	1932	73	3.3	68	4.9	ns	Immediate	CANB 783440
	*Luzula acutifolia* subsp. *nana* Edgar^†^	Rush	16-Feb-11	1957	93	3.3	93	1	ns	Immediate	CANB 797746
Liliaceae	*Astelia alpina* var. *novae-hollandiae* Skottsb.^∧^	Herb	4-Feb-10	1880	77	3.3	76	12.9	ns	Postponed	CANB 792157
	*Astelia psychrocharis* F.Muell.^†^	Herb	4-Feb-10	1830	63	8.8	80	3.5		Staggered (A2)	CANB 792156
	*Herpolirion novae-zelandiae* Hook.f.	Herb	3-Mar-11	1758	53	3.3	0	0	^***^	too low to score	CANB 797834
Poaceae	*Agrostis muelleriana* Vickery	Grass	2-Mar-11	2041	83	3.3	79	2.9	ns	Staggered (B2)	CANB 798298
	*Deschampsia cespitosa* (L.) P.Beauv.	Grass	24-Feb-09	1743	83	8.8	96	2.4		Staggered (A2)	CANB 783436
	*Poa costiniana* Vickery	Grass	30-Mar-11	2109	73	6.7	75	4		Immediate	CANB 798459
	*Poa hiemata* Vickery	Grass	2-Mar-11	2042	83	8.8	65	4.3	ns	Immediate	CANB 798300
	*Rytidosperma alpicola* (Vickery) Connor and Edgar	Grass	5-Feb-09	1747	77	3.5	78	5.6		Immediate	CANB 783601
	*Rytidosperma alpicola* (Vickery) Connor and Edgar	Grass	2-Mar-11	2041	100	0	88	5.4	ns	Immediate	CANB 798297
	*Rytidosperma nudiflorum* (P.Morris) Connor and Edgar	Grass	15-Feb-11	1925	97	3.3	96	1.6	ns	Immediate	CANB 797742
Plantaginaceae	*Plantago glacialis* B. G. Briggs, Carolin and Pulley	Herb	16-Feb-11	1953	93	3.7	82	3.4	ns	Immediate	CANB 797747
Ranunculaceae	*Psychrophila introloba* (F.Muell.) W. A. Weber	Herb	5-Feb-10	1941	96	3.7	98	1.2		Postponed	CANB 792160
	*Ranunculus acrophilus* B. G. Briggs^†^	Herb	6-Jan-10	2039	57	27	76	4.7		Immediate	CANB 786947
	*Ranunculus clivicola* B. G. Briggs	Herb	29-Jan-10	1742	75	6.9	95	2		Staggered (B2)	CANB 792145
	*Ranunculus dissectifolius* F.Muell. ex Benth.^†^	Herb	24-Mar-09	1747	68	17	84	3.4		Postponed	CANB 783454
	*Ranunculus dissectifolius* F.Muell. ex Benth.^†^	Herb	27-Jan-10	1752	48	15	86	6		Postponed	CANB 792148
	*Ranunculus graniticola* Melville	Herb	6-Jan-10	1734	81	9.2	89	4.3		Staggered (A2)	CANB 786945
	*Ranunculus gunnianus* Hook.	Herb	5-Jan-10	1943	97	3.3	81	1.2	^**^	Postponed	CANB 786944
	*Ranunculus muelleri* Benth.	Herb	28-Jan-10	1955	74	16	96	2.4		Staggered (A2)	CANB 786950
Scrophulariaceae	*Euphrasia alsa* F.Muell.^†^	Herb	16-Feb-11	2047	93	6.7	2.3	1.3	^***^	too low to score	CANB 797754
Stackhousiaceae	*Stackhousia pulvinaris* F.Muell.	Mat	8-Apr-09	2085	67	8.3	4	1.6	^***^	too low to score	CANB 782543
Thymalaceae	*Pimelea axiflora* subsp. *alpina* (Benth.) Threlfall	(Sub) shrub	4-Jan-09	1616	47	11	23	4.8	ns	Postponed	CANB 771063

**Figure 2:**
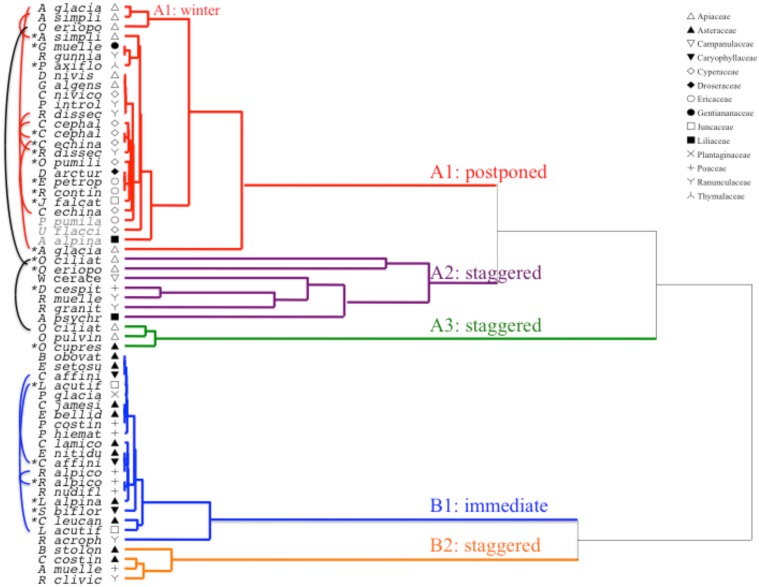
**Cluster analysis dendrogram based on time to cumulative germination percentiles (20, 40, 50, 60, 80, and 95%)**. Species indicated by genus initial and first part of species name. Asterisks mark collections that were banked prior to testing. Fine arcs connect duplicate collections to compare effect of banking on germination. Symbols denote different families (see legend). Collections shown in gray text are those that were exposed to two cycles of the experiment.

*Cluster refers to germination strategy, see **Figure 2**. Herbarium accession number in final column*.

† *Indicates endemic species (Costin et al., 2000)*.

∧ Indicates species that were cycled through the experiment for a second “year.”

P < 0.05, 0.01, and 0.001 shown as ^*^, ^**^, and ^***^ respectively.

Phenology of seed germination was investigated by mimicking temperature regimes that alpine seeds experience in situ, post-dispersal, in an artificially shortened progression of seasons. Seeds were imbibed throughout the experiment thus results indicate potential for germination when water is not limiting. Air and soil temperature data (Figure 1) were used to guide and validate incubator temperature regimes, though the logistical contraints of working with incubators precluded incorporation of the fluctuations inherent to natural conditions. Germination tests used eight replicates of 25 seeds per collection, sown into 9 cm diameter plastic Petri dishes containing 1% plain water-agar. Petri dishes were sealed using Parafilm to avoid agar desiccation, before being placed in germination incubators (Thermoline Scientific, Melbourne, NSW, Australia). Half of the dishes were wrapped in aluminum foil to exclude light and provide an indication of light requirements for germination. Each of the four replicates per light treatment per collection was placed on a different shelf within the same incubator and location within that shelf was re-arranged weekly. All replicates of each collection were moved through the following simulated thermoperiods: 8 weeks at 25/15°C (day/night temperature) → 2 weeks at 20/10°C → 2 weeks at 10/5°C → 10 weeks at constant 4–5°C → 2 weeks at 10/5°C → 2 weeks at 20/10°C → 6 weeks at 25/15°C. Thus, one cycle of the entire experiment mimicked temperatures reminiscent of summer, early autumn, late autumn, winter, early spring, late spring, and summer, and lasted 32 weeks in total. The abbreviated year was considered reasonable because accessions generally had ceased further germination in a given treatment prior to being shifted to the next “season”. A 12/12 h light/dark photoperiod was provided throughout by fluorescent tubes (> 90 μmol m^−2^ s^−1^ at the center of each shelf), and in spring, summer and autumn temperature regimes light coincided with the warm temperature period of the day. Germination, defined as radicle emergence by more than 1 mm, was scored every 7 days, and germinated seeds and seedlings were subsequently removed from the dishes. Germination of foil-wrapped plates was assessed as above but under very low green light.

Following one experimental cycle (32 weeks) most collections were terminated (see supplemental info for exceptions), having exhibited zero germination for at least 2 weeks at this time. Following termination, all remaining intact seeds were dissected with a scalpel under a microscope. Seeds with a firm, fresh endosperm and embryo were deemed viable and seeds empty of an embryo were deducted from the total when calculating percentage germination for each collection. We calculated an index of the light requirements for germination for each species as a fraction of germination in the dark treatment at the time that cumulative germination percentiles of 50 and 95% were reached in the alternating light/dark treatment (see below).

### Ecological correlates of germination strategy

We compiled a database of species' functional traits and geographic measures from published data to assess correlations with germination strategy. The database included the following continuous traits: average seed mass, light requirement for germination (see above), specific leaf area (fresh area/dry mass), individual leaf area, individual leaf dry weight, and height at maturity. The minimum, average and maximum collection elevations for the species were determined from all herbarium records in the Australian Virtual Herbarium, and the elevation at which the specific seed collections used in the study was made was also included as continuous traits. Growth form was scored as one of three categories: graminoid, herb, or shrub. We also included a binary trait: whether the seeds were endospermic or not.

The trait data were collected from a variety of sources including the Australian Virtual Herbarium (http://avh.chah.org.au/), the Australian National Herbarium specimen information register (https://www.anbg.gov.au/cgi-bin/anhsir), Kosciuszko Alpine Flora (Costin et al., 2000), the New South Wales Flora (Harden, 1990), and The comparative internal morphology of seeds (Martin, 1946). Seed mass and specific leaf area were obtained from individual field collections used in the germination trials. Seed mass was obtained by weighing four lots of 10 seeds. SLA was determined based on five fully expanded leaves from separate individuals per species. These were collected in the field, scanned on a flatbed scanner, dried at 60°C to constant weight and weighted to the nearest 0.000 g. Where insufficient seed was available, or specific leaf area was not available, data were obtained from other ANBG collections of the same species or from other data sources (S. Venn, unpublished data). Whether or not a seed was endospermic was determined from drawings of the seed in the ANGB seed bank records in consultation with ANBG seed bank staff. The full database and sources is available on request from the authors.

### Data analysis

Germination patterns were assessed visually and using a cluster analysis in PC-ORD for Windows, version 6 (MJM Software Design, Gleneden Beach, OR, USA). The analysis used a Euclidean distance matrix and a nearest neighbor joining algorithm. Data points for the cluster analysis were the time (weeks) to specific cumulative germination percentiles (t20, t40, t50, t60, t80, and t95%) for each collection (averaged across the 4 dishes). Thus, a collection that germinated early in the experiment would have short times to percentiles up to t80% for example, whereas a collection that postponed germination until temperatures increased following the simulated winter would have long times to these percentiles. More complex germination patterns, such as slow, steady germination or bursts of germination, could also be detected, and would be indicated by evenly spaced times or times clustered at the start and end of the experiment, respectively. This method was used as a way to impose an objective assessment of germination pattern, independent of temperature per se.

A one-way analysis of variance (ANOVA) was carried out for the replicate plates of each collection to assess whether there was a significant difference between percentage viability (as determined by the TZ test), and final percentage germination. Further general linear models were used to assess whether the clusters resulting from the cluster analysis differed in viability or final germination percentage.

Associations between ecological traits and germination strategy clusters were tested in two ways. Firstly, we tested for mean differences between clusters using ANOVA with cluster as a fixed effect and ecological correlates as the response variables, or using replicated G-tests for endospermy and growth form. Secondly, because germination and other functional traits are highly likely to be influenced by evolutionary history, we assessed these correlations using phylogenetic regressions. A phylogeny was constructed for the study species using Phylocom Phylomatic and the bladj packing in R (Webb et al., 2008; R Core Team, 2013). We assessed the presence of phylogenetic structure in variables using the phylosig or phylo.d (discrete or binary variables) function of the Phytools package in R (Revell, 2012). Phylogenetic regressions were conducted using the pgls function in Caper (Orme et al., 2013). Analyses were conducted in a structured way reflecting the clustering revealed in PC-Ord (see below). Initially we included four germination strategies (postponed, immediate, and staggered categories within each of the preceding), then three (lumping all staggered species and comparing to postponed and intermediate) and finally we assessed differences between the two broadest strategies, postponed and intermediate.

## Results

### Seed viability compared to final germination

Overall, seed viability and final germination percentage were high. Mean TZ-estimated viability across all collections was 80 ± 1.7%, with more than two thirds of collections exhibiting more than 75% viability (Table 1). There was no difference in the quality of collections across the 3 years (mean viability in 2009, 2010, and 2011 was 79 ± 2.8, 80 ± 2.7, and 81 ± 3.7%, respectively), indicating that the banking of seeds collected in 2009 did not lessen their viability.

Germination that exceeded or was not significantly lower than TZ-estimated viability was achieved for 45 of the 54 species (One-Way ANOVA: P>0.05, Table 1). Of the remaining nine species, final germination of four species was very low (< 5%), despite good viability (ca. 55–94%), and thus we were unable to draw any conclusions about the germination strategies of these species. Failure to germinate could reflect either that our treatments did not alleviate dormancy or that the seeds were not fully mature at collection. Cut tests at the end of the experiment indicated that, in all cases, the majority of un-germinated seeds contained a healthy, imbibed embryo. Interestingly, 34 species achieved germination that was greater than the TZ-estimated viability (Table 1), suggesting that the TZ test commonly underestimated seed viability.

### Germination clusters

**Figure 3:**
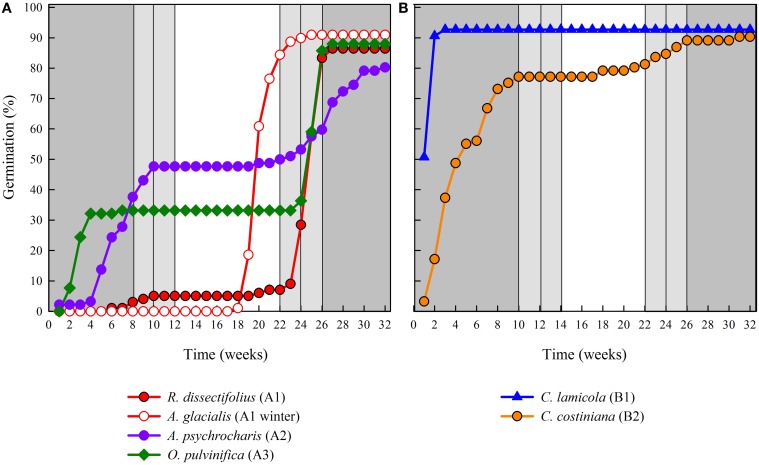
**Cumulative percentage germination of one representative species from each germination cluster**. **(A)** cluster A, species that postponed germination, **(B)** cluster B, species that germinated immediately. Note that staggered germination patterns are nested within the above clusters. Incubator temperature regimes are represented by shading (see Figure 1).

Cluster analysis revealed two major clusters (A and B), with sub-structure in each (Figure 2). For the majority of species (n = 21 species from 10 families), germination was postponed until during a period of winter temperatures (5°C day/night) or until temperatures were raised to 10/5°C following winter (A1 and A1:winter, Figures 2, 3A). Alternatively, germination began immediately at 25/15°C (n = 17 species, six families, seen in cluster B1, Figures 2, 3B). For the majority of these species, germination rate was fast; 50% of the total germination (t_50_) occurred in less than 3 weeks (Supplementary Figure 1).

Within each of these broad classes, however, there were clear sub-clusters reflecting germination that was staggered over time i.e., a proportion of the seed lot germinated before exposure to winter temperatures while the remainder germinated after winter. Staggered germination occurred in 13 species from six families and occurred primarily when daytime temperatures exceeded 10°C (Figures 3A,B). These staggered germinators were broken into three groups in the cluster analysis. Cluster A2 with seven collections showed substantial germination both before and after the cold temperature period. Where germination ceased prior to the temperatures being changed we concluded that the lack of further pre-winter germination was not driven by temperature (A3). In contrast, germination of much of the A2 group appeared to be halted only when temperatures were reduced to 10/5°C. Cluster B2 contained a further four species which exhibited substantial germination early in the experiment and went on to achieve a relatively small proportion of their total germination after temperatures were returned to above 5°C (Figure 3B).

Compared to the relatively minor variation among species within clusters A1 and B1, the staggered clusters also show much greater differentiation among species. Individual curves for all species are shown in Supplementary Figures 1i–vii. For more specific detail on germination strategy see Appendix B in Supplementary Material.

### Ecological correlates of germination strategy

**Figure 4:**
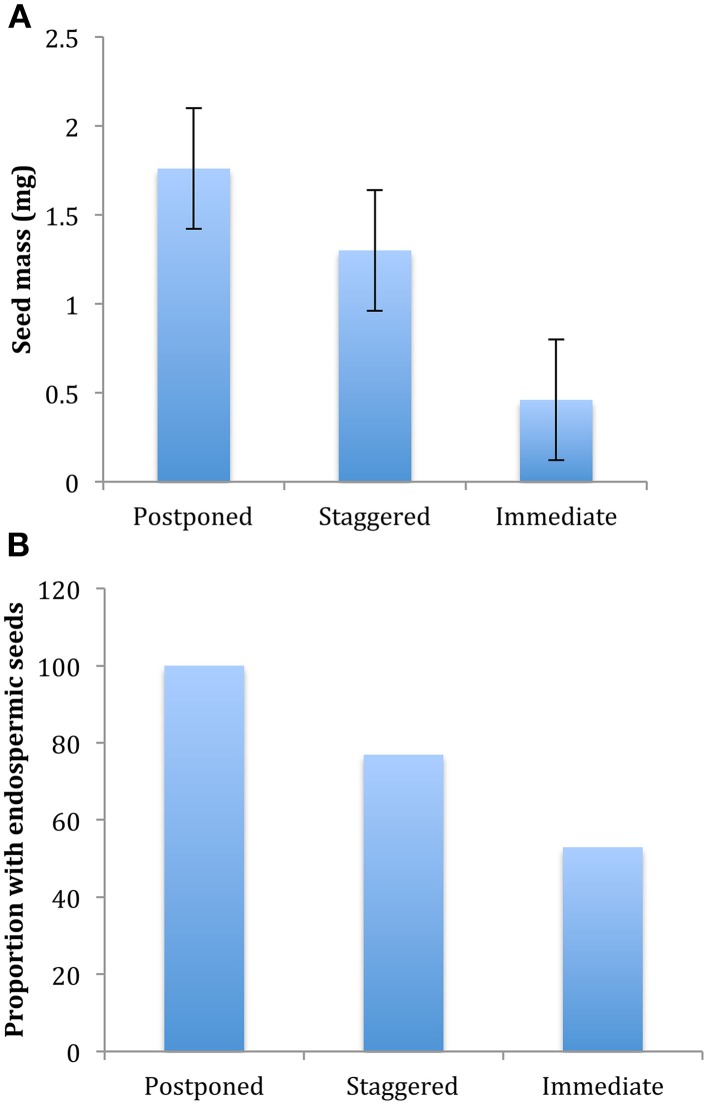
**Differences in (A) average seed mass and (B) proportion of species with endospermic seeds among species with postponed (cluster A1), staggered (combination of cluster A2, A3, and B2) and immediate germination (cluster B1) strategies**. Differences among strategies are significant at P ≤ 0.05, see Table 2.

Of all the ecological correlates that we considered, only those directly associated with seed traits were significantly correlated with germination strategy regardless of whether we assessed the correlation at the level of two major clusters (postponed vs. immediate germination), or at the level of three or four clusters, or whether we accounted for phylogenetic structure or not (Table 2). Species with postponed germination had heavier seeds and were more likely to be endospermic compared to those of species that germinated immediately (Figure 4). Species with staggered germination were intermediate in seed mass and endospermy. However, not all seed characteristics were correlated with germination strategy: there was no correlation between light requirements for seed germination and strategy. Contrary to our expectations, species with higher specific leaf area (potentially indicative of higher growth rates) were not more likely to exhibit immediate germination. Likewise, species with higher average elevation or smaller elevation ranges were not more likely to exhibit postponed germination.

**Table 2:** **Incidence of phylogenetic structure in, and correlates of, germination strategy**.

**Trait**	**Lambda or D value**	**Phylogenetic structure**	**PGLS phyolenetic correlation with germination**		
			**4 Clusters**	**3 Clusters**	**2 Clusters**
Germination strategy^a^	1.532	0.000	na	na	na
Seed mass	1.527	0.000	**0.1098**	**0.008036**	**0.1104**
Endospermic/Non^b^	−9.030	0.000	0.003366	**0.01715**	**0.004963**
Height	1.070	0.022	0.1276	0.4391	0.01948
SLA	< 0.0001	1.000	0.7886	0.2082	0.1998
Surface area	< 0.0001	1.000	0.9141	0.4042	0.7755
Leaf dry weight	1.783	0.020	0.6367	0.2903	0.6017
Average elevation	< 0.0001	1.000	0.6521	0.5852	0.5954
Range	1.227	0.013	0.5775	0.8864	0.63
Light requirement 50%	1.532	0.000	0.1738	0.5999	0.2943
Light requirement 95%	1.007	0.180	0.8247	0.5494	0.9611
Growth form (woody/non)	−0.690	1.000	0.1135	0.8466	0.1499

*Bold indicates significance in non-phylogenetic analyses, yellow cells indicate phylogenetic structure or significance in PGLS regressions*.

a *Germination strategy shows phylogenetic structure at all levels of clustering*.

b *Phylogenetic structure for endospermy and woodiness assessed with Phylo-D for binary value. Probability values for D reflect probablity of pattern resulting from random (no phylogenetic) structure*.

## Discussion

Germination in alpine habitats was historically deemed to be environmentally controlled, with winter snow insulating seeds against potential germination cues and rendering dormancy unnecessary (Billings and Mooney, 1968). However, our results support more recent evidence of dormancy mechanisms being common among alpine species both in Australia (Hoyle et al., 2013; Sommerville et al., 2013) and elsewhere around the world (Cavieres and Arroyo, 2000; Schutz, 2002; Shimono and Kudo, 2005; Mondoni et al., 2009; Schwienbacher et al., 2011). Dormancy would appear to play a significant role in controlling the *in situ* timing of germination of many Australian alpine species, acting to delay, postpone, or slow the rate of germination and thus potentially conferring risk-averse regeneration strategies in a harsh and variable environment. Perhaps most striking of our germination results was the proportion of species for which germination strategy varied within a seed collection for a given species suggesting that both dormant and non-dormant characteristics were exhibited within the same seed collection of these species. Notably, germination strategies were generally highly conserved within families and also correlated with elements of seed anatomy: mass and endospermy. Germination strategies were, however, independent of other ecologically significant functional traits of the mature plant.

### Variation in germination strategy

Seeds of nearly half of the species studied appeared unable to germinate until they were exposed to a cool, wet period (constant 5°C), suggesting that cold stratification alleviated a physiological dormancy mechanism. Postponing germination until the following spring may enable seedlings to avoid establishing over or before the harsh winter, while also optimizing the short forthcoming growing season. In contrast the opportunistic germination exhibited by the species that germinated immediately may provide a selective advantage when the risk of winter seedling mortality is low, by enabling plants to flower earlier the following spring or at a larger size (Donohue, 2002).

To date, there has been little published evidence of intra-specific variability in dormancy mechanisms within alpine seed collections such as would explain the staggered germination strategies observed here. Germination staggered over time may also be explained by varying levels of seed maturity among individuals in the population at the time of collection, and/or could reflect a dimorphic strategy within a single plant associated with position on the plant or timing of development.

Evolutionary bet-hedging is often evoked to explain the diversified strategy in seed germination characteristics (Simons, 2011), such as that demonstrated by the collections with staggered germination. If, however, a diversified strategy results in both increased average fitness and a reduction in variance (e.g., by reducing competition among siblings), then it does not constitute bet-hedging. Likewise, if the apparent diversification reflects differences among, not within, individuals then it is not bet-hedging (Starrfelt and Kokko, 2012). Investigations into alpine seedling dispersal patterns and tolerance of frost and snow melt, paired with studies of determinants of germination strategy variation within species, may help explain germination phenology of the species exhibiting a staggered strategy and may indicate whether they are likely to represent bet-hedging strategies.

### Ecological correlates of germination strategy

Our examination of the relationship between germination strategy and other ecologically important traits demonstrated that germination traits were correlated with other seed traits but not with vegetative traits. Sommerville et al. (2013) found that Australian alpine herbs with non-endospermic seeds were more likely to be non-dormant at dispersal. Our work supports that finding and further indicates that smaller seeds are more likely to be among the immediate germinators. Although Sommerville et al. (2013) did not posit why endospermy may be associated with dormancy, we suggest that placement of reserves in cotyledons may improve early growth rate and establishment in immediate germinating species. Our results did not indicate any correlation between light requirement and dormancy, although light requirements did vary among species. There are a variety of other seed traits that buffer extinction risk in variable environments, for example seed dispersal mechanisms and seed longevity in the soil seed bank (see Tielborger et al., 2012 for a review). At this stage we cannot say whether those traits would show correlations with germination strategy or not.

We further asked whether germination strategy would be correlated with ecologically important non-reproductive traits, such as specific leaf area, or plant height (Westoby, 1998; Wright et al., 2004). In particular we posited that the immediate germinating species might show trait values indicative of faster growth rates (using SLA as a proxy) as might be necessary for establishment in the short period between dispersal and the onset of winter. However, we found no such correlations. Whether physiological and growth traits of seedlings at early establishment stages might show different patterns than do adult vegetative traits is a question worthy of consideration, but beyond the scope of the present work.

Finally, we examined whether species from higher elevations where growing seasons are generally shorter might be more likely to exhibit dormancy but there was no association between either elevation range, collection elevations, or the average elevation of collection for the species as a whole and germination strategy. Together these results indicate that while the evolution of germination strategy is closely linked to ecologically important seed traits, it comprises a largely independent axis to vegetative or distributional characteristics.

### Implications for alpine plant communities in a changing climate

Australian alpine ecosystems, like alpine areas around the world, are under threat from climate change combined with changes in fire frequency, land use patterns, influx of invasive species, and the impacts of increased human visitation. Warming associated with climate change is occurring more rapidly above the treeline than at lower elevations, and alpine areas are predicted to continue to experience above average warming in the future (Kullman, 2004). The capacity for continued regeneration via seed under novel conditions is likely to play a significant role in the response of alpine plants to climate and associated changes. In addition, altered disturbance regimes are likely to require an increased role of restoration and rehabilitation in the region. The insights provided here are therefore crucial to conserving and managing alpine systems under change; they can help inform our predictions of how study species may be affected, form the basis of seed propagation plans for these species, and be used to guide future investigation into alpine seed germination strategies with identifiable conservation and management outcomes.

Changes in temperature are likely to affect the dormancy status of seeds both pre- and post-dispersal, consequently altering and/or disrupting germination strategies and resulting in shifts in current germination phenology (Hoyle et al., 2008b). For alpine plants, climate warming may lead to a shift from spring to autumn emergence, driven primarily by changes in seed dormancy status and resulting in major implications for species currently adapted to emergence in spring (Mondoni et al., 2012). Further research into how changes in temperatures that seeds experience post-dispersal, reduced duration of snow cover, and increased frequency of extreme temperature events will affect germination are required in order to predict how species with dormancy will respond to climate change. For those alpine species that germinate in autumn, prior to frost and snowfall, our results raise the question of how seedlings of these species will cope with the predicted reduction in precipitation and increased frequency of a-seasonal frost events.

Finally, our results raise important questions regarding variation within species, particularly in association with predicting the impact of climate change or selecting which seeds to utilize for restoration. Germination strategies may vary within species depending on population, flowering time, seed mass, parental investment, climate, elevation, and/or maternal environment and this variation may reflect genetic, epigenetic, and/or environmental factors (Cochrane et al., 2015). Increasing elevation accounts for variation in germinability and/or dormancy status of several alpine species (Billings and Mooney, 1968; Dorne, 1981; Cavieres and Arroyo, 2000) but see (Hoyle et al., 2014). Reduced dormancy status can result from early termination of seed development resulting from environmental stresses (Steadman et al., 2006; Hoyle et al., 2008b). Examination of variation in germination strategies within species may reveal the potential for species to cope with, and adapt to, changes in climate, thus, playing an important role in determining future survival and species distributions and providing important insight for management and conservation.

## Conclusions

Our approach has revealed widespread occurrence of dormancy in the Australian alpine flora and demonstrates that germination strategies vary within a seed collection, as well as between collections. Further, we have shown that while dormancy strategy is phylogenetically conserved and correlated with seed mass and endospermy, it is largely independent of vegetative traits and range characteristics. While one cannot extrapolate directly from our results how climate and associated changes will affect the alpine flora, these results can form the basis for design of propagation plans and further experimentation on the impact of changing climatic regimes. In particular, we advocate further research into understanding the causes of within-species variation in germination strategy and the role this may play in the ability of species to withstand predicted climate change.

## Data accessibility

Full germination data and trait database will be lodged on DRYAD and with the Atlas of Living Australia.

### Conflict of interest statement

The authors declare that the research was conducted in the absence of any commercial or financial relationships that could be construed as a potential conflict of interest.
