# Supplementary material for: Seed germination strategies: an evolutionary trajectory independent of vegetative functional traits
Source: Front Plant Sci. 2015 Oct 12;6:731. doi: 10.3389/fpls.2015.00731 (PMC4600905; doi:10.3389/fpls.2015.00731)
Supplement: Supplementary file 1 [file DataSheet1.DOCX]

# *Supplementary materials*

# Authors:

Gemma L Hoyle^1^, Kathryn J Steadman^2,^ Roger B Good^3^, Emma J McIntosh^1^, Lucy M E Galea^1^ and Adrienne B Nicotra^1^*

**Affiliations:**

^1^Department of Evolution, Ecology and Genetics, Research School of Biology, Australian National University, Canberra, ACT, Australia.

^2^School of Pharmacy and Queensland Alliance for Agriculture and Food Innovation, The University of Queensland, QLD, Australia.

^3^Australian National Botanic Gardens, Canberra, ACT 2601, Australia, and Fenner School of the Environment, Australian National University, Canberra, ACT, Australia

**Corresponding author:**

Adrienne Nicotra

Department of Evolution, Ecology and Genetics,

Research School of Biology,

Australian National University,

Canberra, ACT, 2601 Australia.

**Contents:**

**Appendix A: Additional detail on methods**

**Appendix B: Additional detail on germination strategies**

**SI Figure 1.**

Cumulative percentage germination of each species tested in the germination experiment grouped according to cluster analysis. Panels are: i) A1, ii) A1 winter group, iii) A1 species that were exposed to 2 cycles of the experiment, iv) B1 species, v) A2, vi) A3, and vii) B2 species. Duplicate collections are not included unless banking was associated with a change in cluster assignment. Incubator temperature regimes are represented by shading (see Fig. 1).

# Appendix A: Additional detail on methods

## Background:

If freshly collected, mature, viable, water permeable seeds fail to germinate under a range of realistic *ex situ* temperature and moisture conditions, dormancy (often referred to as primary or innate dormancy) can be justifiably diagnosed. One approach to investigating germination strategies under ecologically relevant experimental conditions is by subjecting imbibed seeds to a series of temperature regimes designed to mimic the sequence of seasons that seeds are exposed to *in situ*, post-dispersal; similar to a ‘move-along’ experiment ([Baskin and Baskin, 2004](#_ENREF_4)), or ‘germination phenology study’ ([Albrecht and McCarthy, 2006](#_ENREF_1)). Such experimental designs can be well replicated without requiring excessive amounts of seeds, which allows research to be carried out on species with limited seed production/availability, including valuable conservation collections. This approach has been used in a small number of studies to uncover germination requirements ([Hidayati et al., 2005](#_ENREF_21);[Albrecht and McCarthy, 2006](#_ENREF_1);[Merritt et al., 2007](#_ENREF_34);[Mondoni et al., 2012](#_ENREF_38)), but is by no means commonly adopted.

For logistical reasons alpine germination studies have often been limited in their scope. For example, Schwienbacher *et al*. ([2011](#_ENREF_51)) attempted to classify the dormancy type of 28 Austrian alpine species, but only investigated a single temperature regime (25/10°C, 16/8 hours light/dark), which initiated significant germination of only nine species. Likewise, Shimono and Kudo ([2005](#_ENREF_52)) examined the germination response of 27 alpine species from the Taisetsu Mountains of northern Japan, but warned that their *ex situ* germination conditions were simplified and lacked the seasonal variation of the natural environment. Sommerville *et al*. ([2013](#_ENREF_56)) examined a wide range of stratification durations and germination temperatures for 19 Australian alpine species with the explicit goal of optimising germination under controlled conditions. In contrast, Mondoni *et al*. ([2012](#_ENREF_38)) exposed seeds of eight Italian alpine species to a series of temperatures that mimicked seasonal changes in the laboratory and concluded that germination occurred primarily in spring, after seeds had experienced autumn and winter temperatures.

We focussed on Australian alpine herb field species because these are considered vulnerable to climatic fluctuations ([Hoyle, 2013](#_ENREF_22)), particularly the temperature rises expected to dramatically decrease snow cover and increase growing season length in coming decades. For example, mean temperatures for the Australian alpine in 2050 are projected to exceed those of 1990 by between +0.6 and +2.9 °C with projections of up to 60% decrease in snow cover by 2050 ([Hennessy et al., 2003](#_ENREF_20)), and current data suggest actual increased in temperature may exceed these early projections. A better understanding of the innate germination strategies of alpine species will reveal potential consequences of shortened winters and extended growing seasons on regeneration of alpine plants via seed and will contribute to development of appropriate restoration and management plans for these threatened communities.

## Detailed methods

Mature seeds of 54 species from 16 families and 37 genera were collected between January and April 2009, 2010 and 2011 (see Table 1 for full names and authorities. Twenty-two species were collected in 2009, 19 additional species and eight duplicate collections were added in 2010, and an additional 10 species and three duplicate collections were made in 2011 (Table 1). All 11 duplicate collections were of species already collected in 2009. Vouchers were lodged at the Australian National Herbarium, Canberra). In total the species represented more than a quarter of the Australian alpine angiosperm flora ([Costin et al., 2000, see Table 1](#_ENREF_12)).

In all cases, mature seeds close to the point of natural dispersal were collected from 20 or more plants per population. Seeds were collected from populations where at least some individuals had already begun dispersing seeds. No more than 10% of seeds from any given population were removed at one time. Dispersal units (fruits, capsules or naked seeds), were collected by hand, and placed in paper envelopes. Dispersal units were kept dry and below 20^o^C and returned to the laboratory within three days of collection. Upon returning to the laboratory, non-seed material (including fruits, capsules and plant material) was removed from each collection by hand or by rubbing collections though progressively smaller sieves. *Astelia psychrocharis* F.Muell., *Astelia alpina* var. *novae-hollandiae* Skottsb*.*, *Plantago glacialis* B.G.Briggs, Carolin & Pulley and *Pentachondra pumila* (J.R.Forst. & G.Forst.) R.Br. seeds were removed from fleshy fruits by hand.

Experimentation upon 2010 and 2011 seed collections began almost immediately post-collection. These seeds were stored at *ca*. 15% relative humidity and 15°C for no more than seven days before being sown in experimental conditions (see below). In contrast, collections made in 2009 were banked prior to use in 2010 following international seed-banking procedures ([Smith et al., 2003](#_ENREF_55)). These seeds were stored at *ca*. 20°C in paper envelopes in air-tight containers suspended over silica gel until they reached 15% equilibrium relative humidity (3 – 5 % moisture content) as determined by a Rotronic Hygropalm (AwVC-D10, Bassersdorf, Switzerland). Each collection was then placed in an aluminium envelope that was heat-sealed and stored at -20°C in storage freezers (ThermoScan 2000, Thermoline Scientific, Melbourne, NSW, Australia) for 8 - 11 months. Prior to experimentation, each 2009 collection was removed from storage freezers and allowed to return to room temperature (*ca*. 20°C) for 24 hours before being opened and the seeds sown.

The viability of all collections was estimated prior to sowing in experimental germination conditions using the tetrazolium chloride (TZ) staining technique ([International Seed Testing Association, 2003](#_ENREF_28)). Three replicates of ten seeds per collection were hydrated in Petri dishes containing 1% plain water-agar for 24 hours at room temperature (*ca*. 20°C), before being scarified at a distance from the embryo axis and placed in 1% TZ solution in a dark, 30°C oven for 24 hours. Seeds were then cut in half and embryos examined. Only uniformly stained red/dark pink embryos were considered ‘viable’ ([Hoyle and PrometheusWiki contributors, 2012](#_ENREF_26)).

Our germination method followed elements of the ‘move along’ design, however, as the object of this experiment was to determine the germination of seeds under ecologically relevant conditions rather than laboratory conditions, cold only, warm only and cold-first treatments were not deemed biologically appropriate. The addition of these treatments would also have involved the use of excessive numbers of harvested seeds from natural populations. Furthermore, as rain free periods of more than 10 days rarely occur in the Australian alpine, we presumed that seeds were likely to be sufficiently hydrated for germination at any time post-dispersal and were not likely to require ‘warm, dry after-ripening’ or ‘wet/dry cycling’.

Time permitted us to put three species collected in 2010 through two experimental cycles (a total of 64 weeks) because either seeds were still germinating at the conclusion of one cycle (*Astelia alpina* var. *novae-hollandiae*  (Liliaceae)), or final germination following one cycle was significantly lower than the TZ-estimated seed viability (*Pentachondra pumila* (Ericaceae) and *Uncinia flaccida* S.T.Blake (Cyperaceae)).

# Appendix B: Additional detail on germination strategies

## Seed banking:

Note that we included both fresh and previously banked seed in our experiment. The cluster analysis enabled us to assess the effect of banking on germination pattern for seeds of 11 species that were investigated both pre- and post-banking. For eight of the 11 duplicated species, banking seeds immediately post-harvest did not alter the cluster assignment, although both final germination percentage and finer details of germination response varied between the collections. For example, final germination of previously banked *Luzula acutifolia* subsp. *nana* Edgar (Juncaceae) seeds was lower than that of fresh seeds, but not significantly lower than the viability of the collection as determined by the TZ test (one-way ANOVA: *P* < 0.449, Table 1). *Oreomyrrhis eriopoda* (DC.) Hook.f. (Apiaceae) was one case in which the banked collection had a different enough germination pattern to move the species between clusters in the dendrogram: a proportion of the previously banked *Oreomyrrhis eriopoda* seeds were able to germinate immediately when exposed to summer temperatures (following a lag time of 6 - 7 weeks), whereas germination of all the fresh seeds was postponed until temperatures were reduced to 5^o^C. In *Oreomyrrhis ciliata* Hook.f., banking resulted in a greater proportion of seeds germinating immediately. Finally, fresh seeds of *Gentianella muelleriana* subsp. *alpestris* (L.G.Adams) Glenny (Gentianaceae) reached only 1% germination (data not shown), despite going twice through the simulated year cycle, whereas 74 ± 3.8% of previously banked *Gentianella muelleriana* subsp*. alpestris* seeds germinated following exposure to cold temperatures (5^o^C, Table 1). When the duplicated collections were compared there were no significant differences in viability or final percentage germination between banked and fresh seeds (results not shown). In no case did banking shift a collection between the two major clusters: postponed versus immediate germination. Thus, the effects of banking on germination pattern were minor.

Banked and freshly harvested seeds of eight species for which duplicates were examined generally exhibited the same germination response to temperature, albeit with minor differences. As we were unable to germinate the fresh *Gentianella* seeds at all, we conclude that banking reduced the dormancy status of these seeds ([Turner and Merritt, 2009](#_ENREF_60)). Likewise, in the other two cases in which germination strategy differed between banked and fresh seed, banking was associated with a shift in, or to a pattern of, staggered dormancy such that there was an increase in the proportion of seeds that germinated immediately. Thus, banking had only minor effects on germination strategy and these effects were always in the direction of reducing dormancy status.

## Detailed discussion of germination strategy results

The germination strategies uncovered in this study of alpine herb field species included postponed germination until after ‘winter’, immediate germination, and a staggered strategy which included varying proportions of both immediate and postponed germination. Postponed germination was the most common strategy and suggests that seeds of these species had dormancy at dispersal. The evidence points to physiological and/or morphological dormancy mechanisms that are alleviated via cold stratification. Postponing germination until after winter means that seedling establishment and growth take place during the warmer spring and summer months ([Probert, 2000](#_ENREF_43)). In contrast, seeds of species in the next most common strategy appeared able to germinate immediately, at temperatures reminiscent of those they are dispersed into, without the need for a cold stratification pre-treatment, suggesting that they are non-dormant at dispersal. Regardless of timing, germination of most species occurred when temperatures exceeded 5^o^C; the exceptions being three Apiaceae species that commenced germination at winter-like temperatures. Perhaps most striking was the proportion of species for which germination strategy varied within a seed collection for a given species; these species exhibited staggered germination suggesting that both dormant and non-dormant characteristics were exhibited within the same seed collection of these species.

To the best of our knowledge, this is the first attempt to examine seed germination phenology in response to sequential seasonal temperature regimes of such a significant proportion of any alpine flora. By moving seeds through a series of temperature regimes designed to mimic the progression of seasons post-dispersal we achieved *ex situ* germination of almost one quarter of the entire Australian alpine suite of angiosperms ([as described by Costin et al., 2000](#_ENREF_12)). Note that our approach did not include certain treatments that have been incorporated into previous ‘move-along’ designs ([Hidayati et al., 2005](#_ENREF_21);[Albrecht and McCarthy, 2006](#_ENREF_1)), such as holding seeds at constant temperatures for the entire period or starting seeds at the winter portion of the cycle (i.e. with a cold stratification without prior warm stratification). We focussed on using conditions that were ecologically realistic in our assessment of germination phenology, rather than investigating dormancy and germination requirements *per se*. Although the experimental approach was relatively long-term, 32 weeks per cycle, maintenance and data collection were minimised, as were the number of seeds used per species, which allowed a diverse range of species to be investigated without compromising valuable seedbank accessions. Test conditions culminated in less than 12 months. The resulting data provides significant insight into innate germination strategies. Given the mimicking nature of our experimental design, and the use of fresh, mature seeds collected *in situ*, we consider it reasonable to interpret the resulting germination patterns with regard to *in situ* germination phenology.

Our results indicate that fresh seeds of many species, including several endemics, can be germinated successfully *ex situ*, both pre- and post-banking, often without the need for any pre-treatment. Reliable germination protocols will improve propagation and aid efforts to revegetate disturbed areas, while insights into germination phenology will guide *in situ* season of use and germination expectations over time.

### Species with dormancy:

Seeds of nearly half of the species (21 species (and 5 duplicates) representing ten families) studied appeared unable to germinate until being exposed to a cool, wet period (constant 5°C), suggesting that cold stratification alleviated a physiological dormancy mechanism. Postponing germination until the following spring may enable seedlings to avoid establishing over or before the harsh winter, while also optimising the short forthcoming growing season. This was the most common germination pattern exhibited by species tested. Seeds of *Aciphylla glacialis* (F.Muell.) Benth., *Aciphylla simplicifolia* (F.Muell.) Benth. and *Oreomyrrhis eriopoda* (Apiaceae) began to germinate at cold temperatures reminiscent of those found beneath the snow during winter (4 - 5^o^C, 12/12 hours, light/dark, Cluster A1: winter in Fig. 2, SI Fig 1.ii). The remaining 18 species (9 families) in cluster A1 began significant germination following the winter treatment, when day temperatures reached or exceeded 10^o^C. These species included a further two Apiaceae species, four of the five Cyperaceae species studied, and the only representatives of Droseraceae and Gentianaceae in the Australian Alps (Fig. 2, SI Fig 1,i).

Seeds of three species exhibited low germination and were maintained in the experiment for a second cycle: *Pentachondra pumila* (Ericaceae) exhibited significant germination (> 5%) following a second winter treatment and *Astelia alpina* var. *novae-hollandiae* (Liliaceae) and *Uncinia flaccida* (Cyperaceae) exhibited additional germination following a second winter (SI Fig 1.iii).

Seeds of all the Apiaceae, Cyperaceae and Ericaceae species considered (n=8, 4 and 3 respectively) would appear to be dormant at dispersal. Dormancy is thought to be more common in Cyperaceae than any other alpine family ([Schutz, 2000](#_ENREF_49)), and *Carex ferruginea* and *Carex frigida* from the Swiss Alps also postpone germination following dispersal ([Schutz, 2002](#_ENREF_50)). In addition, most of the Ranunculaceae species tested (6 of 7) exhibited dormancy.

Seeds of three species did not begin to germinate until seeds had experienced a second period of winter temperatures or achieved greater germination after a second rotation of the experiment. Thus, it is possible that the duration of cold or the minimum temperature (5°C) in this study was inadequate for dormancy alleviation or these species require extended periods for dormancy alleviation. These species, as well as the remaining four that failed to germinate, deserve further examination.

Since all seeds received both warm and cold stratification, it is unclear whether dormancy alleviation in some of the alpine species required warm-plus-cold stratification, as reported for some non-alpine species ([Baskin et al., 2002](#_ENREF_5)), or simply just the cold stratification. A slow germination rate and/or a lag time to germination may indicate that the germination temperature window widened with gradual dormancy loss during the initial warm stratification, until it overlapped with test condition temperatures, at which point germination could commence ([Vleeshouwers et al., 1995](#_ENREF_62)). We consider it possible that at least some of these alpine species’ seeds respond to warm stratification given that they are dispersed onto a warm, moist soil surface during summer (10-15°C in this study), and that there is often an extended period between seed maturation and the first frosts/snowfall.

A lag time to germination can also be indicative of an underdeveloped embryo that requires time to mature before germination can commence, often termed morphological dormancy ([Nikolaeva, 1977](#_ENREF_39)). Indeed, morphological dormancy is common among species of Apiacaeae, Ranunculaceae and Liliaceae species ([Baskin and Baskin, 2001](#_ENREF_3)), including the alpine herb *Psychrophila introloba* (F.Muell.) W.A.Weber (Ranunculaceae, Wardlaw *et al*., 1989). Low temperature requirements vary among the different stages of dormancy alleviation in seeds with morpho-physiological dormancy ([Phartyal et al., 2009](#_ENREF_42)). *Aciphylla* *glacialis* (Apiaceae) embryos are under-developed inside seeds at the time of dispersal and grow at both 5°C and 10/5°C, however, only the seeds at 5°C go on to germinate (Hoyle et al 2014)).

For the majority of species tested, late spring/summer temperatures were most conducive for post-winter germination. In the field this timing would enable seedlings to avoid solar radiation stress during snowmelt and freeze-thaw situations common to the alpine zone in early spring ([Körner, 2003b](#_ENREF_30)). In contrast, *Aciphylla glacialis, Aciphylla simplicifolia* and *Oreomyrrhis eriopoda* (Apiaceae) seeds began to germinate after approximately 6 weeks in the cold incubator (~5°C), suggesting that *in situ* germination may occur during winter when snow cover insulates seedlings from freezing temperatures ([Drescher and Thomas, 2013](#_ENREF_17)). It is arguable that germinating during winter would give seedlings space and resource advantages during the growing season that seedlings emerging in late spring may lack.

### Immediate germinators

The second most common germination pattern was germination almost immediately after being sown (Cluster B1 in Fig. 2). Seeds of 17 species (and 3 duplicates) from 6 families germinated in temperature conditions indicative of the warm growing season into which they are dispersed (25/15°C, 12/12 hours, light/dark, Fig. 3b). Species exhibiting this germination pattern included nine of the 11 Asteraceae species, five of the seven Poaceae species and both Caryophyllaceae species investigated. For the majority of species, germination rate was fast; 50% of the total germination (t_50_) occurred in less than 3 weeks (data not shown). The only Ranunculaceae species that fell into this group, *Ranunculus acrophilus* B.G.Briggs, exhibited a lag time to germination of 4 - 5 weeks, which sets it apart somewhat from the other members of this cluster (SI Fig 1.iv).

Australian alpine seeds are dispersed in late summer/autumn, after which immediate, rapid and synchronous germination appears to be the regeneration strategy of many species, particularly within the Asteraceae and Poaceae. Such opportunistic germination may provide a selective advantage when the risk of winter seedling mortality is low, by enabling plants to flower earlier the following spring or at a larger size ([Donohue, 2002](#_ENREF_14)).

### The staggered strategy

Finally**,** germination of a further 13 species (two of which were duplicates) from 6 families was staggered over time, occurring at warm temperatures, both before and after exposure to 10 weeks at winter temperatures (Fig 3a,b, Fig 1.v-vii). These staggered germinators were broken into three groups in the cluster analysis. Cluster A2 with 7 collections showed substantial germination both before and after the cold temperature period. For some of these species the halt in germination coincided with the onset of colder temperatures while for others it preceded the temperature change. Collections in Cluster A3 had a small amount of germination early in the experiment but the majority of germination occurred after the 5^o^C period had ended. In all three of the A3 collections germination ceased well before the temperatures had been decreased to ‘autumn’ conditions. Because germination ceased prior to the temperatures being changed we concluded that the lack of further pre-winter germination was not driven by temperature. In contrast, germination of much of the A2 group (e.g. *Ranunculus muelleri* Benth., and *Astelia psychrocharis*) appeared to be halted only when temperatures were reduced to 10/5^o^C. Cluster B2 contained a further four species which exhibited substantial germination early in the experiment and went on to achieve a relatively small proportion of their total germination after temperatures were returned to above 5^o^C (Fig 3b). Compared to the relatively minor variation among species within clusters A1 and B1, the staggered clusters also show much greater differentiation among species (individual curves for all species are shown in SI Fig 1i-vii).

It is also possible that seeds of staggered species that germinated before winter were not entirely non-dormant at dispersal; if cold stratification over winter were found to widen the temperature conditions in which germination can occur seeds may be referred to as having ‘conditional’ or ‘relative’ dormancy when dispersed ([Vegis, 1964](#_ENREF_61)). Indeed, cold stratification has been found to reduce the minimum temperature at which other alpine/tundra seeds can germinate, through eliciting changes in seed dormancy status ([Densmore and Zasada, 1983](#_ENREF_13);[Shimono and Kudo, 2005](#_ENREF_52);[Mondoni et al., 2009](#_ENREF_37)). Such changes in dormancy status may explain some of the staggered germination patterns we saw, thus, dormancy may be even more common among alpine species than our data and other published work indicate.

**REFERENCES IN APENDICES**

Baskin, C. C., Zackrisson, O., and Baskin, J. M. (2002). Role of warm stratification in promoting germination of seeds of *Empetrium hermaphroditum* (Empetraceae), a circumboreal species with a stony endocarp. *Am. J. Bot.* 89, 486–493. doi: 10.3732/ajb.89.3.486

Drescher, M. & Thomas, S.C. (2013) Snow cover manipulations alter survival of early life stages of cold-temperate tree species. *Oikos,* **122,** 541-554.

Hennessy, K., Whetton, P., Smith, I., Bathols, J., Hutchinson, M. & Sharples, J. (2003) The impact of climate change on snow conditions in mainland Australia. CSIRO, Aspendale VIC.

Hidayati, S.N., Baskin, J.M. & Baskin, C.C. (2005) Epicotyl dormancy in Viburnum acerifolium (Caprifoliaceae). *The American Midland Naturalist,* **153,** 232-244.

Hoyle, G.L., Cordiner, H., Good, R.B. & Nicotra, A.B. (2014) Effects of reduced winter duration on seed dormancy and germination in six populations of the alpine herb Aciphyllya glacialis (Apiaceae). *Conservation Physiology,* **2,** cou015-cou015.

Hoyle, G.L. & PrometheusWiki contributors (2012) PrometheusWiki, Seed viability, TZ testing.

Körner, C. (2003) *Alpine Plant Life: Functional plant ecology of high mountain ecosystems*. Springer, Verlag, Berlin, Heidelberg, New York.

Merritt, D.J., Turner, S.R., Clarke, S. & Dixon, K.W. (2007) Seed dormancy and germination stimulation syndromes for Australian temperate species. *Australian Journal of Botany,* **55,** 336-344.

Nikolaeva, M.G. (1977) Factors controlling the seed dormancy pattern. *he physiology and biochemistry of seed dormancy and germinatio* (ed. A. Khan), pp. 51-74. North-Holland Publishing Company, New York.

Phartyal, S.S., Kondo, T., Baskin, J.M. & Baskin, C.C. (2009) Temperature requirements differ for the two stages of seed dormancy break in *Aegopodium* *podagraria* (Apiaceae), a species with deep complex MPD. *American Journal of Botany,* **96,** 1086-1095.

Probert, R.J. (2000) The role of temperature in the regulation of seed dormancy and germination. *Seeds: The Ecology of Regeneration in Plant Communities* (ed. M. Fenner), pp. 261-292. CAB International.

Smith, R., Dickie, J., Linington, S., Pritchard, H., and Probert, R., (eds.). (2003). *Seed Conservation: Turning Science into Practise*. London: Kew Publishing.

Schutz, W. (2000). Ecology of seed dormancy and germination in sedges (*Carex*). *Perspect. Plant Ecol. Evol. Syst.* 3, 67–89. doi: 10.1078/1433-8319-00005

Turner, S.R. & Merritt, D.J. (2009) Seed germination and dormancy. *Plant germplasm conservation in Australia: strategies and guidelines for developing, managing an utilizing ex situ collections* (eds C.A. Offord & P.F. Meagher), pp. 87-108. Australian Network for Plant Conservation Inc., Canberra.

Vegis A (1964) Dormancy in higher plants. *Annual Review of Plant Physiology,* **15,** 185-224.

**SI Figure 1.**

**
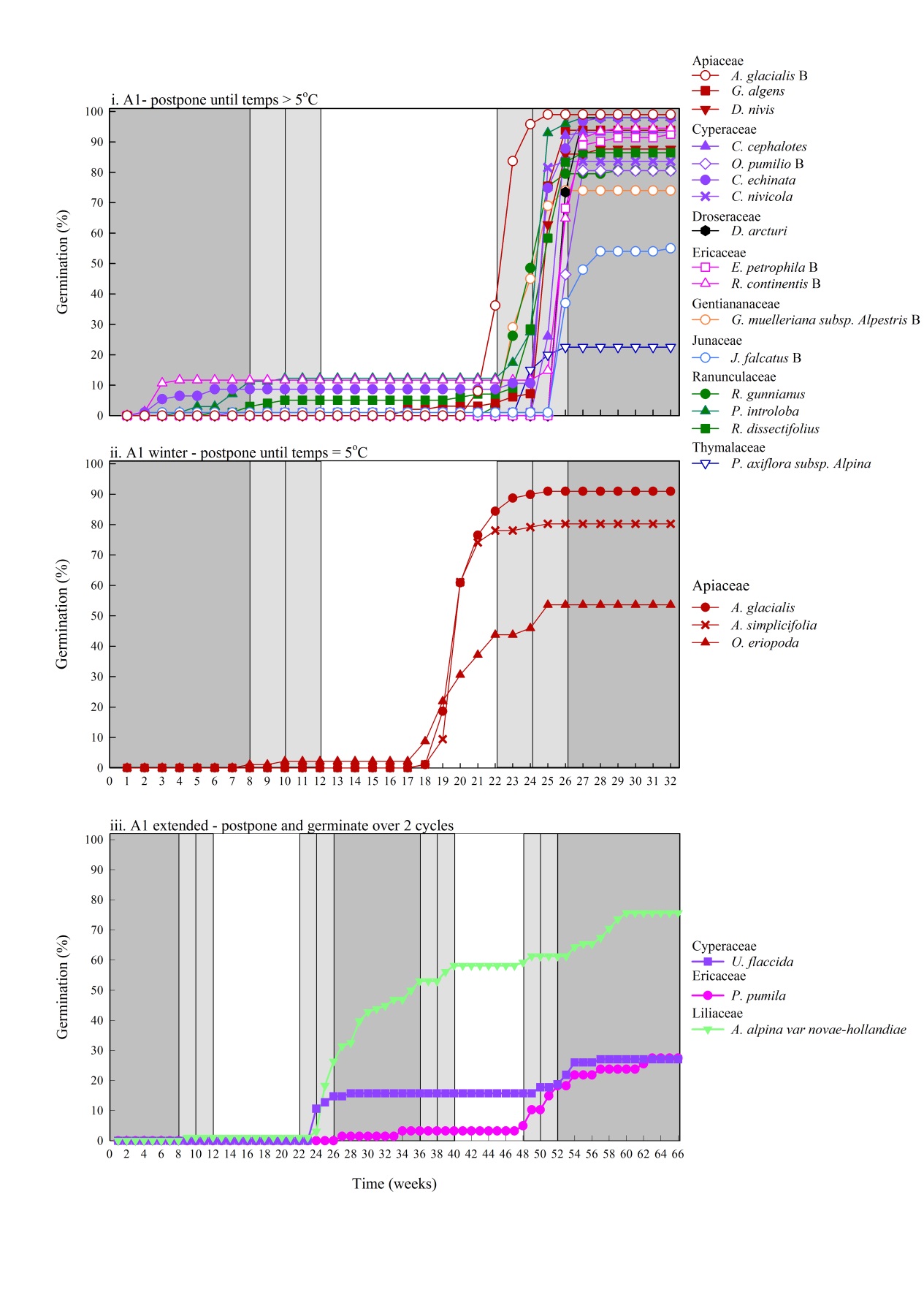
**Cumulative percentage germination of each species tested in the germination experiment grouped according to cluster analysis. Panels are: i) A1, ii) A1 winter group, iii) A1 species that were exposed to 2 cycles of the experiment, iv) B1 species, v) A2, vi) A3, and vii) B2 species. Duplicate collections are not included unless banking was associated with a change in cluster assignment. Incubator temperature regimes are represented by shading (see Fig. 1).

**
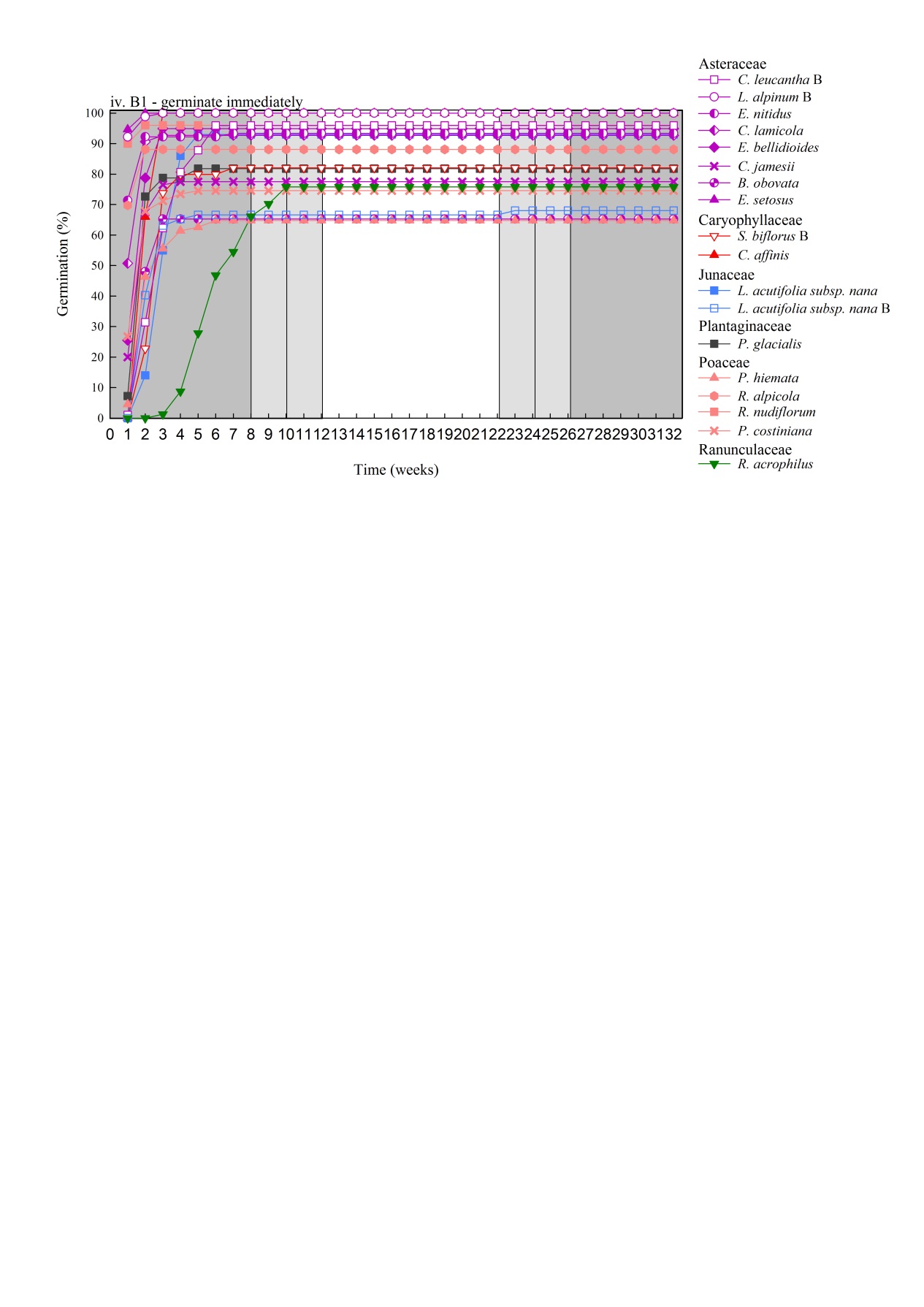
**

**
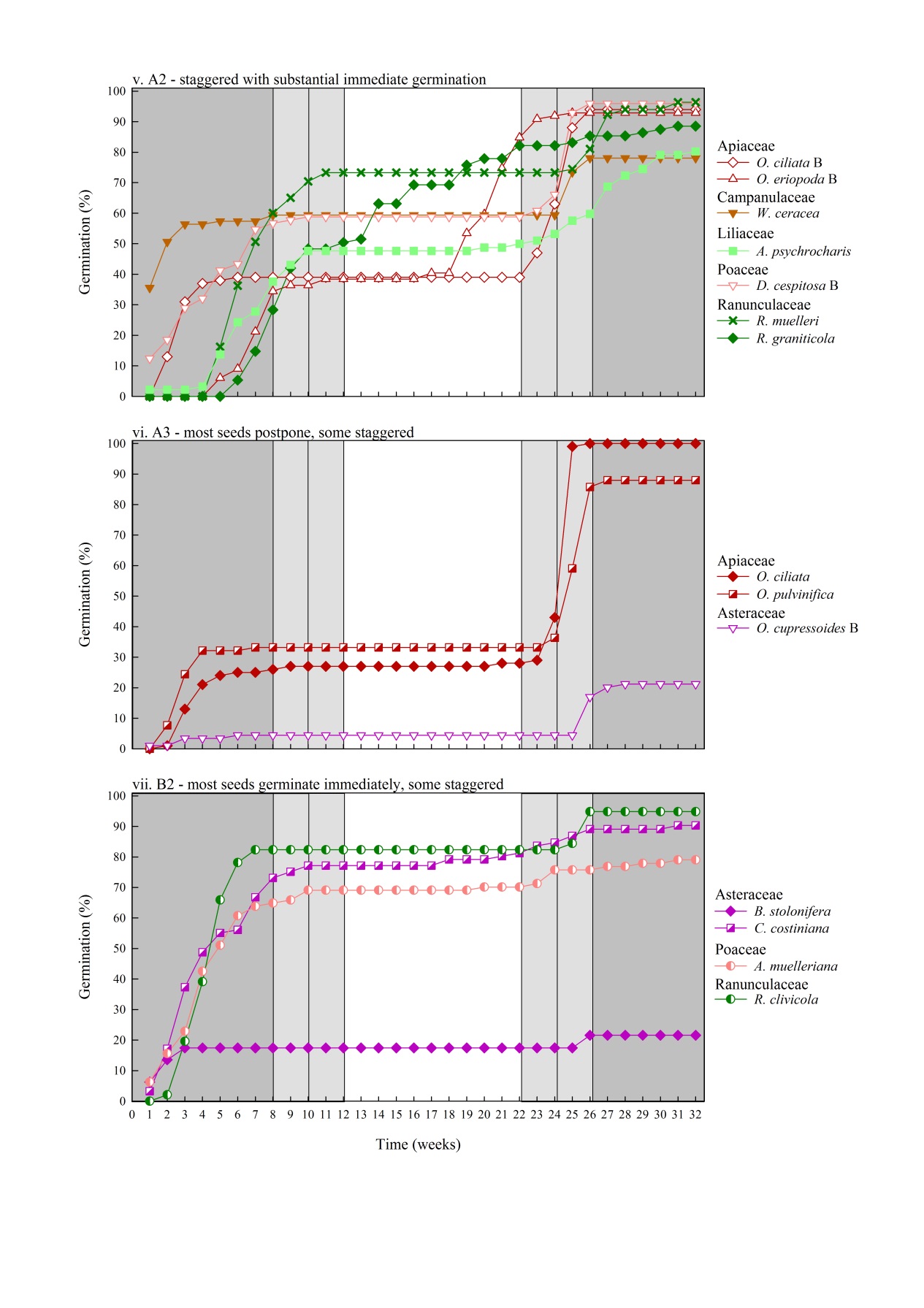
**
