# Supplementary material for: Seed germination strategies: an evolutionary trajectory independent of vegetative functional traits
Source: Front Plant Sci. 2015 Oct 12;6:731. doi: 10.3389/fpls.2015.00731 (PMC4600905; doi:10.3389/fpls.2015.00731)
Supplement: Supplementary file 2 [file Table1.DOCX]

| Family | Species | Growth form | Date collected | Elevation  (m a.s.l.) |
| --- | --- | --- | --- | --- |
| Apiaceae | *Aciphylla glacialis* (F.Muell.) Benth. | Herb | 11 Mar 2009 | 2164 |
|  | *Aciphylla glacialis* (F.Muell.) Benth. | Herb | 17 Feb 2010 | 1937 |
|  | *Aciphylla simplicifolia* (F.Muell.) Benth. | Herb | 12 Mar 2009 | 1698 |
|  | *Aciphylla simplicifolia* (F.Muell.) Benth. | Herb | 16 Feb 2010 | 1743 |
|  | *Diplaspis nivis* Van den Borre & M.J.Henwood | Herb | 28 Jan 2010 | 1960 |
|  | *Gingidia algens* (F.Muell.) Dawson | Herb | 16 Feb 2010 | 1740 |
|  | *Oreomyrrhis ciliata* Hook.f. | Herb | 24 Mar 2009 | 1748 |
|  | *Oreomyrrhis ciliata* Hook.f. | Herb | 27 Jan 2010 | 1748 |
|  | *Oreomyrrhis eriopoda* (DC.) Hook.f. | Herb | 24 Feb 2009 | 1743 |
|  | *Oreomyrrhis eriopoda* (DC.) Hook.f. | Herb | 3 Feb 2010 | 1744 |
|  | *Oreomyrrhis pulvinifica* F.Muell. | Herb | 4 Feb 2010 | 1733 |
|  | *Schizeilema fragoseum* (F.Muell.) Domin | Herb | 31 Mar 2011 | 2092 |
| Asteraceae | *Brachyscome obovata* G.L.Davis | Herb | 2 Mar 2011 | 2090 |
|  | *Brachyscome stolonifera* G.L.Davis | Herb | 5 Feb 2010 | 1941 |
|  | *Celmisia costiniana* Max Gray & Given | Herb | 2 Mar 2011 | 2046 |
|  | *Craspedia jamesii* J.Everett & Joy Thomps. | Herb | 1 Feb 2011 | 1730 |
|  | *Craspedia lamicola* J.Everett & Joy Thomps. | Herb | 3 Mar 2011 | 2067 |
|  | *Craspedia leucantha* F.Muell. | Herb | 25 Feb 2009 | 1996 |
|  | *Erigeron bellidoides* (Hook.f.) S.J.Forbes & D.I.Morris | Herb | 29 Jan 2010 | 1730 |
|  | *Erigeron nitidus* S.J.Forbes | Herb | 17 Feb 2010 | 2007 |
|  | *Erigeron setosus* (Benth.) Max Gray | Herb | 4 Feb 2010 | 1928 |
|  | *Leucochrysum albicans* subsp. *alpinum* (F.Muell.) Paul G.Wilson | Mat/Herb | 11 Mar 2009 | 2212 |
|  | *Ozothamnus* sp. (aff. *hookeri*) Sond. | Shrub | 9 Apr 2009 | 1605 |
| Campanulaceae | *Wehlenbergia ceracea* Lothian | Herb | 3 Feb 2010 | 1744 |
| Caryophyllaceae | *Colobanthus affinis* | Herb | 25 Mar 2009 | 2069 |
|  | *Colobanthus affinis* | Herb | 5 Jan 2010 | 1736 |
|  | *Scleranthus biflorus* | Mat/Cushion | 10 Mar 2009 | 1751 |
| Cyperaceae | *Carex cephalotes* | Sedge | 4 Jan 2009 | 1613 |
|  | *Carex cephalotes* | Sedge | 5 Jan 2010 | 1640 |
|  | *Carex echinata* | Sedge | 21 Jan 2009 | 1684 |
| Family | Species | Growth form | Date collected | Elevation  (m a.s.l.) |
|  | *Carex echinata* | Sedge | 3 Feb 2010 | 1742 |
|  | *Carpha nivicola* | Sedge | 3 Feb 2010 | 1730 |
|  | *Oreoblus pumilio* | Sedge | 7 Apr 2009 | 1945 |
|  | *Uncinia flaccida* | Sedge | 3 Mar 2011 | 2047 |
| Droseraceae | *Drosera arcturi* | Herb | 27 Jan 2010 | 1752 |
| Ericaceae | *Epacris petrophila* | Shrub/Subshrub | 26 Mar 2009 | 1745 |
|  | *Pentochondra pumila* | Mat/Subshrub | 17 Feb 2010 | 2096 |
|  | *Richea contientis* | Subshrub | 8 Apr 2009 | 2078 |
| Gentiananaceae | *Chionogentias mulleriana* subsp*. alpestris* | Herb | 24 Mar 2009 | 1748 |
|  | *Chionogentias mulleriana* subsp*. alpestris* | Herb | 30 Mar 2011 | 2109 |
| Juncaceae | *Juncas falcatus* | Rush | 10 Mar 2009 | 1742 |
|  | *Luzula acutifolia* subsp. *nana* | Rush | 25 Feb 2009 | 1932 |
|  | *Luzula acutifolia* subsp. *nana* | Rush | 16 Feb 2011 | 1957 |
| Liliaceae | *Astelia alpina var novae-hollandiae* | Herb | 4 Feb 2010 | 1880 |
|  | *Astelia psychrocharis* | Herb | 4 Feb 2010 | 1830 |
|  | *Herpolirion novae-zelandiae* | Herb | 3 Mar 2011 | 1758 |
| Poaceae | *Agrostis muelleriana* | Grass | 2 Mar 2011 | 2041 |
|  | *Deschampsia caespitosa* | Grass | 24 Feb 2009 | 1743 |
|  | *Poa costiniana* | Grass | 30 Mar 2011 | 2109 |
|  | *Poa hiemata* | Grass | 2 Mar 2011 | 2042 |
|  | *Rytidosperma alpicola* | Grass | 5 Feb 2009 | 1747 |
|  | *Rytidosperma alpicola* | Grass | 2 Mar 2011 | 2041 |
|  | *Rytidosperma nudiflorum* | Grass | 15 Feb 2011 | 1925 |
| Plantaginaceae | *Plantago glacialis* | Herb | 16 Feb 2011 | 1953 |
| Ranunculaceae | *Psychrophila introloba* | Herb | 5 Feb 2010 | 1941 |
|  | *Ranunculus acrophilus* | Herb | 6 Jan 2010 | 2039 |
|  | *Ranunculus clivicola* | Herb | 28 Jan 2010 | 1742 |
|  | *Ranunculus dissectifolius* | Herb | 24 Mar 2009 | 1747 |
|  | *Ranunculus dissectifolius* | Herb | 27 Jan 2010 | 1752 |
|  | *Ranunculus graniticola* | Herb | 6 Jan 2010 | 1734 |
|  | *Ranunculus gunnianus* | Herb | 5 Jan 2010 | 1943 |
| Family | Species | Growth form | Date collected | Elevation  (m a.s.l.) |
|  | *Ranunculus muelleri* | Herb | 28 Jan 2010 | 1955 |
| Scrophulariaceae | *Euphrasia alsa* | Herb | 16 Feb 2011 | 2047 |
| Stackhousiaceae | *Stackhousia pulvinaris* | Mat | 8 Apr 2009 | 2085 |
| Thymalaceae | *Pimelea axiflora* ssp. *alpine* | Subshrub | 4 Jan 2009 | 1616 |
